# Treadmill training augmented with real-time visualisation feedback and function electrical stimulation for gait rehabilitation after stroke: a feasibility study

**DOI:** 10.1186/s42490-019-0020-1

**Published:** 2019-08-22

**Authors:** Chanwit Phongamwong, Philip Rowe, Karen Chase, Andrew Kerr, Lindsay Millar

**Affiliations:** Department of Biomedical Engineering, University of Strathclyde, Glasgow, UK

**Keywords:** Treadmill training, Functional electrical stimulation, Visualisation feedback, 3-dimentional motion capture, Stroke

## Abstract

**Background:**

Stroke rehabilitation often uses the motor relearning concept that require patients to perform active practice of skill-specific training and to receive feedback. Treadmill training augmented with real-time visualisation feedback and functional electrical stimulation may have a beneficial synergistic effect on motor recovery. This study aims to determine the feasibility of this kind of enhanced treadmill training for gait rehabilitation among patients after stroke. A system for dynamic visualisation of lower-limb movement based on 3-dimentional motion capture and a computer timed functional electrical stimulation system was developed. Participants received up to 20-min enhanced treadmill training instead of their over-ground gait training once or twice a week for 6 weeks at Coathill hospital, Lanarkshire, United Kingdom. Number of training sessions attended, and training duration were used to assess feasibility. Ankle kinematics in the sagittal plane of walking with and without functional electrical stimulation support of the pre-tibial muscles were also compared and used to confirm the functional electrical stimulation was triggered at the targeted time.

**Results:**

Six patients after stroke participated in the study. The majority of participants were male (5/6) with a age range from 30 to 84 years and 4/6 had left hemiplegia. All participants suffered from brain infarction and were at least 3 months after stroke. Number of training sessions attended ranged from 5 to 12. The duration of training sessions ranged from 11 to 20 min. No serious adverse events were reported. The computerised functional electrical stimulation to the pre-tibial muscles was able to reduce plantarflexion angle during the swing phase with statistical significance (*p* = 0.015 at 80%; *p* = 0.008 at 90 and 100% of the gait cycle).

**Conclusions:**

It is safe and feasible to use treadmill gait training augmented with real-time visual feedback and computer-controlled functional electrical stimulation with patients after stroke in routine clinical practice.

**Trial registration:**

NCT03348215. Registered 20 November 2017.

## Background

Stroke is a common neurological disease leading to many impairments and disabilities [1, 2]. The loss of or difficulty with walking is one of the most common concerns of stroke survivors. Impairment of motor control are the most common sequelae after stroke affecting approximately two third of stroke survivors [3], and seems to be the major contribution to walking difficulty after stroke. Patients immediately after a significant stroke are often dependent ambulators. Although, most patients after stroke are able to walk after a period of time often involving a rehabilitation programme, many of them do not reach community ambulation levels [4].

To encourage neuroplasticity, stroke rehabilitation often uses the motor relearning concept that requires patients to perform active practice of skill-specific training and to receive feedback [5]. Functional electrical stimulation (FES) is the application of a low-level electrical current to elicit contraction in weak or paralyzed muscles due to upper motor neuron injuries/diseases such as stroke. It is used to perform specific functions; for example, arm/hand control, standing, or walking. It can be used as an assistive device (neuroprosthetic effect) or to help restore or improve patient’s movement during rehabilitation such as drop foot stimulation during the swing phase of stroke survivors in gait retraining [6]. Moreover, there is clinical evidence that FES can encourage motor relearning and neuroplasticity by changing cortical excitability and stimulating cortical reorganization (therapeutic effect) [7]. Because stroke can affect gait performance in both stance and swing phase, multichannel FES (MFES) might have the potential for assisting gait training among patients after stroke. The clinical evidence indicates that MFES improves gait performance among patients with chronic stroke [8, 9]. The use of MFES for acute stroke combined with treadmill training may also be feasible and safe [10, 11] and may enhance acute recovery.

Three-dimensional kinematic motion capture systems (3D-MoCap) are one of the most accurate investigation tools for gait analysis. They can provide joint and segment kinematics, gait parameters, and can determine phases of the gait cycle. Nowadays, due to advanced computer technologies, 3D-MoCap can be used to create dynamic visualisation of lower-limb movement which provide patients after stroke with real-time visual feedback for motor relearning [12, 13]. It can also provide patients with a real-time feedback-controlled treadmill that adjusts continuously the treadmill speed to the patients’ gait speed which is called self-paced treadmill walking [14]. Treadmill training with or without body-weight support has been shown to increase walking speed and capacity but not to achieve greater levels of independent walking. However, treadmill training augmented by real-time visualisation feedback and computer-controlled FES may have a beneficial synergistic effect [15] and may enhance recovery. Hence, the present study aims to develop a 3D-MoCap based MFES system and to determine the feasibility of the treadmill training enhanced with real-time visual feedback and computerised FES for gait rehabilitation among patients after stroke.

## Results

Six patients after stroke participated in the feasibility study. In Table 1, it can be seen that the majority of participants were male (5/6) with an age range of 30 to 84 years and 4/6 had left hemiplegia. All participants suffered from brain infarction and were at least 3 months since stroke. Additionally, walking speed and RMI before and after treatment are given in Table 1. The patients’ feedback from the questionnaire is summarized in Table 2.

**Table 1:** Participant’s characteristics and performance

Participant	Gender	Age (yrs.)	Type of stroke	Hemiplegic side	Time since stroke (month)	Number of training sessions attended/appointed	Training duration (min) ^a^	Walking speed (m/s) B/A	RMI^b^ B/A
1	Male	61	Infarction	Left	6	11/12	15 (8–20)	0.12/0.13	4/8
2	Male	84	Infarction	Left	3	6/6	11 (9–13)	0.44/0.35	9/−
3	Female	30	Infarction	Right	12	11/12	18 (10–20)	0.82/0.74	13/14
4	Male	40	Infarction	Left	4	12/12	20 (10–20)	0.44/0.59	12/13
5	Male	55	Infarction	Right	10	5/6	15 (15–20)	0.19/0.17	10/12
6	Male	47	Infarction	Left	12	5/6	20 (20–20)	0.67/0.62	10/12

*Abbreviation*: *RMI* for Rivermead Mobility Index, *B/A* for Before/After treatment

^a^ Median (Min – Max); ^b^ Higher RMI scores show better mobility (maximum of 15 is possible)

**Table 2:** Results of patients’ feedback on enhanced treadmill training

Statement	P1	P2	P3	P4	P5	P6
The amount of set up time before each session was acceptable.	2	NG	4	5	4	5
Walking with all of the equipment set up was not too cumbersome.	4	NG	3	5	2	5
The treadmill is comfortable to walk on, and easy to get used to.	2	NG	4	5	5	4
Walking with the harness was comfortable and made me feel safe.	3	NG	2	5	2	5
The sessions as a whole were enjoyable.	5	NG	4	5	4	4
The instructions given during the sessions were easy to understand and carry out.	5	NG	4	5	5	4
I still feel motivated to continue with this training program.	4	NG	5	5	5	4
I would recommend treadmill training such as this to other stroke survivors.	4	NG	4	5	5	5

1 = Strongly disagree; 2 = Disagree; 3 = Neutral; 4 = Agree; 5 = Strongly agree

*Abbreviation*: *NG* for Not given

**Fig. 1:**
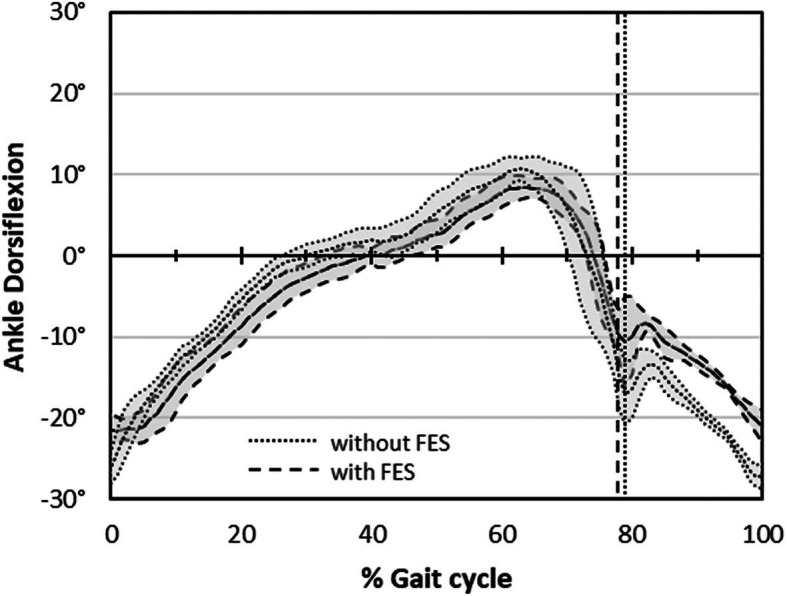
The ankle kinematics when walking with and without FES to the pre-tibial muscles

For the 6 participants, a total of 50 sessions of enhanced treadmill training were carried out. Number of training sessions attended ranged from 5 to 12. The duration of training sessions ranged from 11 to 20 min. However, no participant was able to complete 20-min training without a break. The treadmill was operated in fixed speed for all participants because we found they could not use self-paced mode for their comfortable speed. All participants received FES to the pre-tibial muscles to correct drop foot in all training session. Also, FES to the quadriceps muscles was added for some training sessions of Participant 4 (7 sessions) and 5 (3 sessions). To show the FES was triggered at the targeted time, the ankle kinematics of one participant walking with and without FES for drop foot (0.27 m/s walking speed for both conditions) were compared (Fig. 1). Of nine gait cycles, walking with FES showed less plantarflexion angle than walking without FES during swing phase. The Wilcoxon signed-rank test revealed significant difference at 80% (*p* = 0.015); 90% (*p* = 0.008); and 100% (*p* = 0.008) of the gait cycle.

No serious adverse events were reported. However, Participant 1 reported increased spasticity of the toe flexors in week 4 of treatment programme. FES did not cause this problem, but it could potential raise the patient’s discomfort. Hence, an AFO was used instead of FES for the last 4 sessions for this patient. Participant 3 requested to terminate one session after 14 min of training due to hemiplegic arm pain.

## Discussion

Currently, some treadmills augmented with force sensor or platform have been marketed commercially and the kinetic data (e.g. foot pressure or ground reaction force) is used as visual feedback for gait re-training after stroke [16]. However, to our knowledge the present study is the first to use 3D-MoCap to create dynamic visualisation of lower-limb movement and step length/ratio as real-time visual feedback for treadmill gait training. In addition, the developed FES system is the first to be triggered using real-time 3D-MoCap data while the previous studies on MFES [10, 17, 18] relied on foot switches constructed from force-sensing resistor and placed on the heel (or the heel/toe) which could not be detected the sub-phases of stance or swing. The accuracy and repeatability of the gait events detected by foot switch is also dependent on the placement of the sensors on the foot [19].

According to our results, patients valued the enhanced treadmill training as there was a high attendance rate. Three participants attended all their training sessions whereas three participants could not attend only one session due to personal reason. Some participants were also able to complete 20 min of repetitive training from treadmill. Additionally, positive feedback on the training using the new system was given. All 5 participants who give their feedback stated that they enjoyed the enhanced treadmill training and motivated to continue the training programme. They would recommend enhanced treadmill training to other patients after stroke. However, some patients indicated that walking with the harness made them uncomfortable.

This study was similar to the study of Daly et al. that tested the feasibility of combining MFES with weight-supported treadmill training [10]. The major difference between the two studies was the type of FES electrode and the visualisation. Daly and colleague used intramuscular electrodes which was inserted in operating rooms by a surgeon. This study used surface electrodes which are easy to don and doff in an out-patient clinic and are less invasive. Moreover, the study of Daly et al. did not provide patients with any type of biofeedback during gait rehabilitation.

Although only the feasibility phase of the present study has been completed, preliminary results from the first 6 participants showed some important information. Firstly, it is clear that the 3D-MoCap system can be used during routine treatment in clinical practice. Using cluster markers for the MoCap requires the attachment of only 7 clusters which can be attached over trousers and the subsequent pointer anatomical calibration lasts only 2 min. Importantly, the method was quick enough to provide patients with visual feedback in real time. Secondly, based on the kinematic results of the ankle joint compared between walking with and without FES support on the pre-tibial muscles, it was shown that the developed computerised FES system triggered the FES at the targeted time when using the 3D-MoCap but all participants had slow walking speeds Finally, it can be concluded that treadmill gait training augmented with real-time visualisation feedback and FES is safe and feasible for stroke rehabilitation.

Some limitations were encountered. Firstly, anatomical landmarks on the pelvis are difficult to identify for some participants due to the wearing of the fall prevention harness. This may affect the accuracy of hip joint kinematics. Next, it was difficult to bring patients who needed lifting and handling support into/out of the treadmill because of limited space for the staff within the treadmill. Lastly, because the present study used surface electrical stimulation, some participants experienced cutaneous pain or ankle clonus when high level of stimulation were needed.

## Conclusions

The evidence from this study supports the conclusion that the treatment modality is safe, and that it is feasible to use treadmill gait training augmented with real-time visualisation feedback and FES with patients after stroke in clinical practice. Its effect on motor recovery remain to be determined.

## Methods

### Participants

The study was conducted at Coathill Hospital (NHS Lanarkshire), UK. Adults (over 18 years old) who had suffered a hemiplegic stroke from 1 week to 12 months and were medically stable and fit for gait rehabilitation were eligible to participate in the study. Patients who had severe cognitive impairment; significant hip, knee, or ankle contracture; walking difficulty before stroke; or contraindication for FES (e.g. using cardiac pacemaker or having metal implant under electrode sites) were excluded. Participants were enrolled prospectively, and written consent to participate in the study was obtained. This paper reports the development of the system and its feasibility with 6 participants who finished the training programme. The project is ongoing with a targeted sample size of 20 cases. The study was approved by NHS West of Scotland Research Ethics Committee (Reference: 17/WS/0245) and was registered in ClinicalTrials.gov (Reference: NCT03348215). R&D approval was granted by NHS Lanarkshire (R&D ID: L18006).

### Training programme

All participants received up to 20 min of enhanced treadmill training session instead of their over-ground gait training session once or twice a week for 6 weeks. During training sessions, any ankle foot orthosis they were prescribed was removed. Participants could request pauses in the training session if needed. The treadmill was equipped with front and side bars from which support could be gained if required.

### Research equipment and technology

#### Treadmill and body-weight supported system

**Fig. 2:**
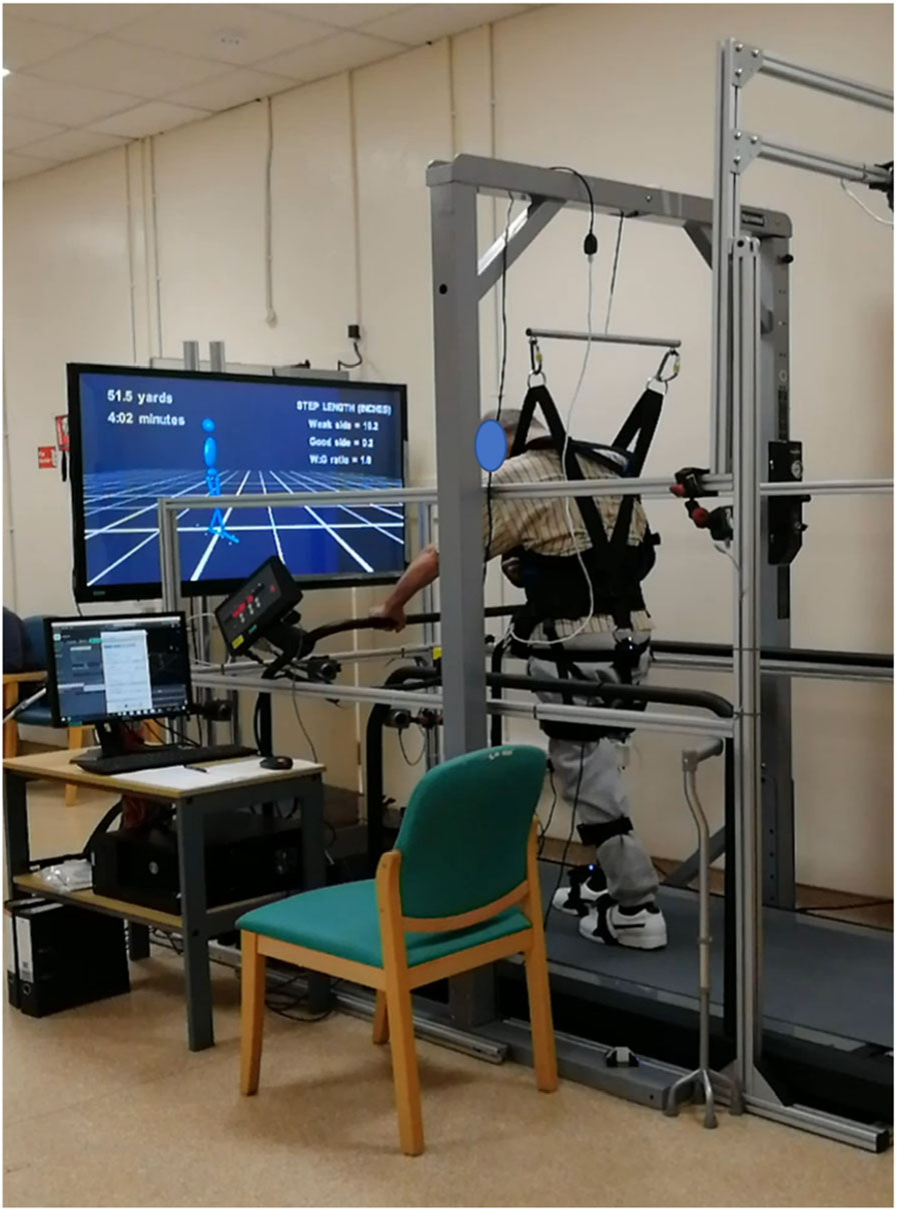
Treadmill and body-weight support system

The study used an N-Mill treadmill (Model: N-Mill 1 N75, ForceLink B.V., Culemborg, Netherlands) that can adjust its speed to match the user in other words operated in self-paced mode using D-Flow software. A harness and pneumatic body-weight supported system (PneuMex) was also provided (Fig. 2). The amount of body-weight support was adjusted to take into account the walking ability of each participant. However, all participants wore the harness attached to the suspension system to prevent falls.

#### 3D-MoCap system

The 3D-MoCap consisted of a 6-camera motion analysis system (Bonita, Vicon Inc., Oxford, UK) which was installed around the treadmill. A cluster based method for gait analysis with pointer anatomical calibration was used [20]. The clusters of markers were placed on the pelvis, both thighs, both shanks, and both feet for the whole training session. Vicon Tracker software (Vicon Motion Systems, Oxford, UK) was used to identify cluster sets as objects, and to stream marker trajectory information into D-Flow (Motekforce Link, Netherlands). A 3D visualisation package D-flow, then, manipulated the input data from the 3D-MoCap using scripts written in the Lua programming language or using the provided D-Flow modules. The D-flow application presented the movement data as an avatar to the subjects on a large TV screen at the head of the treadmill. The anatomical calibration, the calculation of joint kinematics/gait parameters, and the phases of the gait cycle were carried out by D-flow at 60 Hz in real time.

**Fig. 3:**
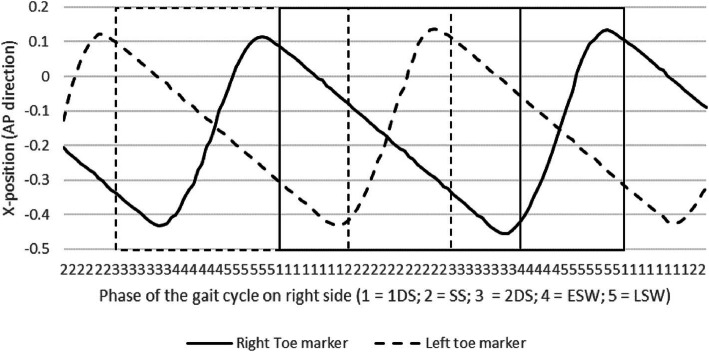
The phase of the gait cycle determined by toe marker trajectory

The study used the toe marker trajectory on both sides to establish the phases of the gait cycle on the hemiplegic side. For treadmill walking, the stance phase was determined when the toe marker moved backward whereas the swing phase was determined when the marker moved forward. In addition, the stance phase was divided into 3 subphases: first double limb support (1DS); single limb support (SS); second double limb support (2DS) while the swing phase was divided in to 2 subphases: early swing (ESW) and late swing (LSW) as shown in Fig. 3.

#### FES

Dual-channel surface electrical stimulators (NeuroTrac® Rehab, Model number: ECS305A) were used in the present study. The electrical impulse was an asymmetrical, rectangular bi-phasic waveform. The commercial stimulator had a pushbutton remote switch to control the stimulation. When the remote switch was connected to the device, the electrical stimulation could be stopped and re-started by pressing the pushbutton switch. In the study, the pushbutton remote switch was removed and replaced with a computerised switch under software control. The stimulators were not modified, and were used in the custom mode (P15 mode) with 300 μs of pulse duration, 30 Hz of pulse frequency, and 0.1 s of ramp up/down. Amplitude (mA) was maximised to reach appropriate muscle function without pain or discomfort. Self-adhesive reusable skin electrodes size 50 × 50 mm were used individually for each subject.

For the FES software switch control system, an Arduino board was used. It had digital pins that could be outputs used to control the stimulators. The remote-control socket of the electrical stimulators was connected to the output pins of the Arduino producing a series of computer-controlled ON/OFF switches. The HIGH and LOW output are then similar to pressing and un-pressing the original remote switch. The Arduino board received real-time information (a number from 1, 2, 3, 4 or 5) from the computer giving the phase of the gait cycle (1 = 1DS; 2 = SS; 3 = 2DS; 4 = ESW; 5 = LSW) as calculated by D-flow. These output values were used to trigger the FES devices at the targeted time.

**Fig. 4:**
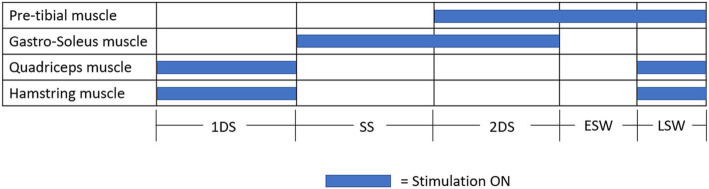
Stimulation pattern of FES

Up to 4 FES devices could be used for: Pre-tibial muscle (FES1) for reducing drop foot problem during SW; Gastro-soleus muscle (FES2) for restraining the tibia’s progression during SS and 2DS; Quadriceps (Vastus Medialis/Lateralis) muscle (FES3) and Hamstring (Semitendinosus/Biceps femoris [long head]) muscle (FES4) for improving hip and knee stability during 1DS (Fig. 4). The treating physiotherapist judged which FES devices were used to support gait retraining.

#### Visualisation feedback

**Fig. 5:**
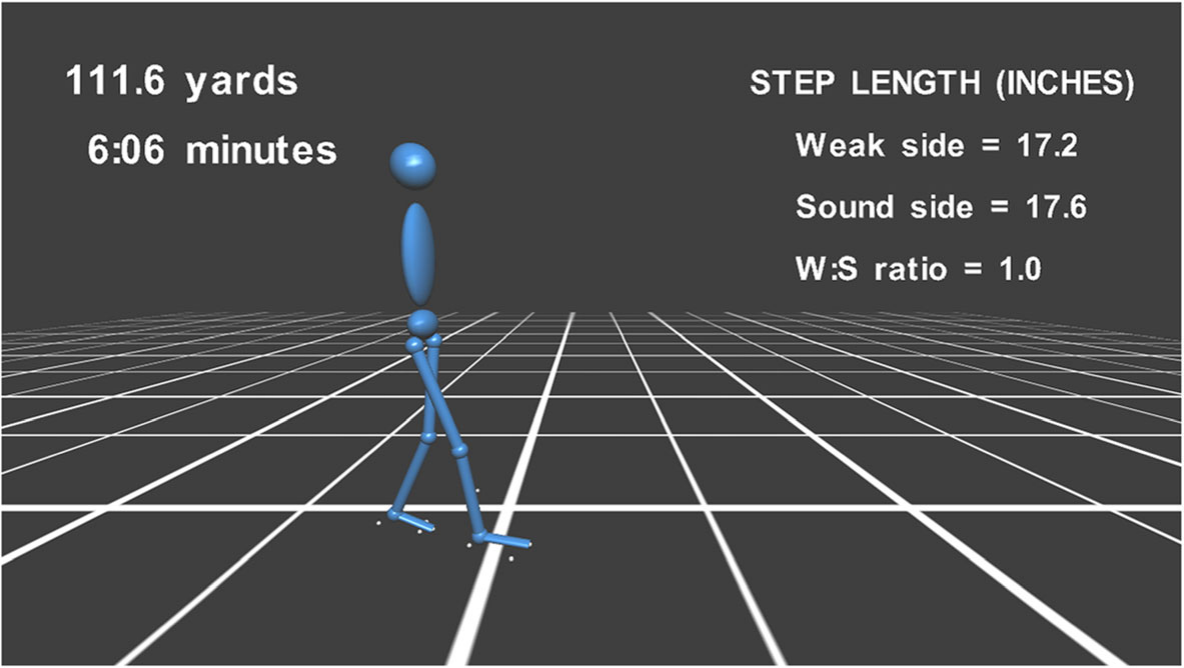
Real-time visualisation feedback

Within the D-flow software, the animation was constructed and displayed in the “DSR” visualisation window. The dynamic visualisation of lower-limb kinematics was developed by linking segments with common joint centres (Fig. 5) [20]. Cylindrical objects were used to create each segment, and spherical objects were placed at each joint centre. A separate head, trunk, and pelvis was included in the Avatar. This visualisation was used for real-time visual feedback. Moreover, step length of both sides and step ratio were calculated and shown on the screen in real time. Step length was the distance in the antero-posterior direction between both toe markers at initial foot contact and step ratio was hemiplegic step length divided by the non-hemiplegic one. The distance covered and time spent walking were also shown on the screen.

### Outcome measures

Number of training sessions attended, and training duration were used to assess the feasibility of enhanced treadmill training. In addition, joint kinematics in the sagittal plane of walking with and without FES support were evaluated to determine whether the developed FES system worked well in clinical practice. Moreover, walking speed from a 10-m walk test and functional mobility assessed by Rivermead Mobility Index (RMI) [21–23] were collected before and after treatment. Patients’ feedback based on a structured questionnaire was collected after patients completed their training programme.

### Statistical analysis

Descriptive statistics were obtained including mean with standard deviation or median with interquartile range for continuous data (e.g. age, time since stroke onset), and number and percentage for categorical data (e.g. gender, hemiplegic side). The statistical difference of continuous outcomes (e.g. gait speed, joint kinematics) between 2 sets of dependent data (e.g. walking with FES and without FES) was examined using Wilcoxon signed-rank test given the small group size.

## Data Availability

The datasets generated during the current study are available from the corresponding author on reasonable request.

## Abbreviations

3D-MoCap: Three-dimensional kinematic motion capture
DS: Double limb support
FES: Functional electrical stimulator
NHS: National Health Service
RMI: Rivermead Mobility Index
SS: Single limb support
SW: Swing
